# Long-term outcomes of patients who rate symptoms of rheumatoid arthritis as ‘satisfactory’

**DOI:** 10.1093/rheumatology/kez497

**Published:** 2019-11-15

**Authors:** James M Gwinnutt, Kimme L Hyrich, Mark Lunt, RAMS Co-Investigators, Anne Barton, Suzanne M M Verstappen

**Affiliations:** 1 Centre for Epidemiology Versus Arthritis, Centre for Musculoskeletal Research, Faculty of Biology, Medicine and Health, University of Manchester, Manchester Academic Health Science Centre; 2 NIHR Manchester Biomedical Research Centre, Manchester University NHS Foundation Trust, Manchester Academic Health Science Centre; 3 Centre for Genetics and Genomics Versus Arthritis, Centre for Musculoskeletal Research, Faculty of Biology, Medicine and Health, University of Manchester, Manchester Academic Health Science Centre, Manchester, UK

**Keywords:** rheumatoid arthritis, epidemiology, disability, cluster analysis, patient acceptable symptom state

## Abstract

**Objectives:**

To describe outcomes of patients with early RA in a patient acceptable symptom state (PASS) at treatment initiation and to identify clusters of symptoms associated with poor outcomes.

**Methods:**

Data came from the Rheumatoid Arthritis Medication Study, a UK multicentre cohort study of RA patients starting MTX. The HAQ, DAS28 and other patient-reported outcome measures (PROMs) were collected at baseline, and at 6 and 12 months. Patients answering yes to the question ‘Is your current condition satisfactory, when you take your general functioning and your current pain into consideration?’ were defined as PASS; patients answering no were defined as N-PASS. Symptom clusters in the baseline PASS group were identified using K-medians cluster analysis. Outcomes of baseline PASS *vs* N-PASS patients and each cluster are compared using random effects models.

**Results:**

Of 1127 patients, 572 (50.8%) reported being in PASS at baseline. Over one year, baseline PASS patients had lower DAS28 (mean difference = −0.71, 95% CI −0.83, −0.59) and HAQ scores (mean difference = −0.48, 95% CI −0.56, −0.41) compared with N-PASS patients. Within the baseline PASS group, we identified six symptom clusters. Clusters characterized by high disease activity and high PROMs, or moderate disease activity and high PROMs, had the worst outcomes compared with the other clusters.

**Conclusion:**

Despite reporting their condition as ‘satisfactory’, early RA patients with high PROM scores are less likely to respond to therapy. This group may require increased vigilance to optimize outcomes.

**Rheumatology key messages**

- Half of RA patients commencing MTX are in an acceptable symptom state, despite receiving no treatment.
- On average, RA patients in an acceptable state have improved outcomes over one year.
- Certain subgroups of RA patients in an acceptable state do poorly, and may require monitoring.

## Introduction

Objectives of disease management for patients with RA include achieving remission, reducing disability and improving quality of life [1, 2]. However, remission is not attainable for all patients [3, 4], and therefore a useful measure of outcome is whether patients have achieved a level of health that is deemed ‘acceptable’ by the patient, the ‘patient acceptable symptom state’ (PASS) [5]. This state relates to the concept of patients feeling ‘well’ as opposed to an improvement in symptoms leading patients to feel ‘better’, measured using the minimal clinically important difference [6].

In previous studies, patients rated moderate levels of disease activity as acceptable. An analysis of patients with established disease [mean (s.d.) disease duration = 7.6 (9.1) years] from the NOR-DMARD cohort showed that the acceptable level of the DAS28 was <4.21 at week 12 and <3.90 at week 52, while for the HAQ the cut-off point was <1.04 for both week 12 and 52 (with cut-off point defined as the 75th centile of scores in patients with self-reported acceptable symptom state) [5]. Despite these moderate levels of disease activity and disability, patients in PASS had lower levels of pain and reported being able to cope with their condition better than patients not in PASS (N-PASS). This was confirmed in a cross-sectional study across 10 European countries where both higher pain and coping scores were associated with reduced odds of being in PASS [pain odds ratio (OR) = 0.80, 95% CI 0.67, 0.96; coping OR = 0.84, 95% CI 0.71, 0.97] [7].

This pan-European study was a cross-sectional analysis of prevalent cases of RA (mean symptom duration 12.6 years). It is likely that patients with established disease have changed their definition of what disease state is acceptable over time, as previous studies have demonstrated differences in illness perceptions between early and established RA, and changes over time for RA and OA [8–10]. There is limited information about PASS in patients with early RA commencing a conventional synthetic DMARD for the first time, such as the prevalence and predictors of PASS in this group. This understanding would allow clinicians to monitor patients who are unlikely to be satisfied with their condition in the future. Furthermore, the group of patients in PASS at baseline is likely to be heterogeneous, with different phenotypes or combinations of symptoms that are deemed ‘acceptable’. Some of these phenotypes may be associated with worse outcomes and these more ‘reticent’ patients could be monitored more intensively.

Therefore, the aims of this analysis were to describe the number of patients in PASS at baseline and 12 months, to compare the outcomes of patients in PASS at baseline over 12 months against those who are not, and to identify common phenotypes of symptoms within patients in PASS at baseline and assess the outcomes of these phenotypes over 12 months.

## Methods

This analysis included patients with RA commencing MTX for the first time. Patients were recruited to the Rheumatoid Arthritis Medication Study (RAMS), a prospective observational cohort study recruiting patients from 38 secondary healthcare centres across the UK, commencing in 2008 [11]. All included patients received a diagnosis of RA or inflammatory arthritis from their rheumatologist and were about to receive their first treatment of MTX at recruitment. Exclusion criteria were: recruited >2 years after their first symptoms, not conventional synthetic DMARD naïve prior to MTX and failed to answer baseline PASS question (supplementary Fig. S1, available at *Rheumatology* online). This study complies with the Declaration of Helsinki. RAMS ethical approval was obtained from the National Research Ethics Service Central Manchester Research Ethics Committee (ref: 08/H1008/25) and all patients gave their written informed consent.

### Assessments

Patients were assessed at baseline, and at 6 and 12 months. Demographics were collected at baseline. A clinician performed a 28 swollen and tender joint count (SJC28/TJC28) at each assessment and a physician global visual analogue scale (VAS) was completed by the consultant. Blood samples were taken at each visit, posted to the study co-ordinating centre in Manchester and stored (−80°C freezers) for future analysis, including: CRP level (mg/l) and baseline RF positivity (latex test, positive cut-off 14 units/ml).

Patients completed a self-reported patient questionnaire at each visit, including the British version of the HAQ – Disability Index [12], a self-reported measure of functional disability. Patients also completed pain, fatigue and global assessment VAS and the Hospital Anxiety and Depression Scale (HADS), calculating separate scores for both anxiety and depression [13].

The DAS28 was calculated at each assessment using the SJC28, TJC28, CRP level and patient global assessment [14, 15].

### Definition of PASS

At each assessment, patients answered the question ‘Is your current condition satisfactory, when you take your general functioning and your current pain into consideration?’ Those who answered yes were defined as in PASS, those answering no were defined as N-PASS.

### Statistical analysis

The baseline characteristics of the patients are displayed using descriptive statistics for the whole cohort and stratified based on PASS status at baseline. Independent associations between baseline variables (age, gender, symptom duration, smoking status, SJC28, TJC28, CRP, HAQ, RF, pain-VAS, fatigue-VAS, patient global-VAS, physician global-VAS, HADS depression, HADS anxiety) and PASS status at baseline were assessed using a logistic regression model. Multiple imputation was used to account for missing data [10 datasets created; missing baseline data ranged from complete to 17.7% missing (RF)].

K-medians cluster analysis was used to identify distinct phenotypes within the group of patients in PASS at baseline. Variables used in the cluster analysis were defined *a priori* based on variables that reflect a range of symptoms relevant to patients with RA and affect quality of life [16]. These variables were: SJC28, TJC28, HAQ, pain-VAS, fatigue-VAS and HADS-depression. The optimum number of clusters was determined using the ‘elbow method’ [17]. Multiple imputation was used to account for missing data. The clustering algorithm was run in each imputed dataset separately. The modal cluster from the 10 imputed datasets was used when reporting descriptive statistics of each cluster. The clusters were numbered based on a severity score (supplementary Table S1, available at *Rheumatology* online). For regression analysis, cluster assignment was allowed to vary between imputed datasets (see supplementary Table S2, available at *Rheumatology* online, for description of agreement in cluster assignment across imputations) [18].

The 6- and 12-month outcomes of the patients are reported using descriptive statistics, stratified by baseline PASS status. The HAQ, DAS28, pain-VAS, fatigue-VAS, HADS-depression and HADS-anxiety scores over the repeated measures were compared between patients in PASS *vs* N-PASS at baseline using random effects models, controlling for age and gender. Being in PASS over follow-up was compared between PASS groups at baseline using Generalized Estimating Equations analysis.

To compare the outcomes of the different clusters of patients in PASS at baseline, the HAQ, DAS28, pain-VAS, fatigue-VAS and HADS-depression of the clusters were compared over the repeated measures using random effects models, controlling for gender and baseline age. Being in PASS at later follow-ups was compared between clusters using Generalized Estimating Equations analysis. Clusters were compared with baseline N-PASS patients using the same methods. All analyses were performed using Stata version 14 (Stata Statistical Software Release 14, 2015; StataCorp, College Station, TX, USA).

## Results

In total, 1127 patients were included in this analysis. These patients had a median age of 60 years [interquartile range (IQR) 50, 69] and 714 (63.4%) were women. The cohort had moderate levels of disease activity and disability [median (IQR) DAS28 = 4.1 (3.2, 5.2); HAQ = 1.00 (0.38, 1.63)] at baseline, and 65.2% were RF+ (Table 1).

**Table 1:** Baseline characteristics for the total cohort and stratified by baseline PASS status

	Total cohort		PASS		N-PASS		
Variable	*N*	Median (IQR)	*N*	Median (IQR)	N	Median (IQR)	*P*
Age, years	1127	60 (50, 69)	572	63 (51, 70)	555	59 (49, 68)	0.0002^a^
Women, *N* (%)	714 (63.4)		343 (60.0)		371 (66.9)		0.017^b^
Symptom duration, months	1127	6 (4, 10)	572	6 (3, 9)	555	7 (4, 12)	0.0016^a^
Smoking status, *N* (%)	1118		570		548		0.5581^c^
Never	433 (38.7)		225 (39.5)		208 (38.0)		
Ex	456 (40.8)		228 (40.0)		228 (41.6)		
Current	229 (20.5)		117 (20.5)		112 (20.4)		
SJC28	1093	4 (2, 9)	550	3 (1, 7)	543	6 (2, 11)	<0.0001^a^
TJC28	1094	5 (2, 12)	550	4 (1, 8)	544	8 (3, 14)	<0.0001^a^
CRP, mg/l	1119	5 (2, 16)	569	4 (2, 11)	550	8 (3, 22)	<0.0001^a^
DAS28 (CRP)	1078	4.1 (3.2, 5.2)	544	3.6 (2.9, 4.5)	534	4.7 (3.8, 5.7)	<0.0001^a^
HAQ	1119	1.00 (0.38, 1.63)	566	0.63 (0.13, 1.13)	553	1.38 (0.88, 1.88)	<0.0001^a^
RF, *N* (%)	928		470		458		0.527^b^
Positive	605 (65.2)		311 (66.2)		294 (64.2)		
Negative	323 (34.8)		159 (33.8)		164 (35.8)		
Pain-VAS	1102	47 (24, 69)	554	28 (14, 48)	548	65 (47, 77)	<0.0001^a^
Fatigue-VAS	1104	49 (22, 72)	558	31 (12, 55)	546	65 (43, 79)	<0.0001^a^
Patient global-VAS	1118	36 (20, 60)	568	26 (15, 46)	550	50 (30, 68)	<0.0001^a^
Physician global-VAS	1094	34 (20, 55)	554	25 (14, 41)	540	48 (29, 64.5)	<0.0001^a^
HADS-depression	1112	5 (2, 8)	565	3 (1, 6)	547	7 (4, 10)	<0.0001^a^
HADS-anxiety	1110	6 (3, 9)	565	5 (2, 7)	545	7 (4, 11)	<0.0001^a^
Taking steroids, *N* (%)							
Oral	259 (23.1)		138 (24.3)		121 (21.9)		0.337^b^
I.m.	264 (24.0)		126 (22.7)		138 (25.4)		0.293^b^

a Mann–Whitney U test. ^b^Chi^2^ test. ^c^Kruskal–Wallis test. HADS: Hospital Anxiety and Depression Scale; IQR: interquartile range; N: number; N-PASS: not in a patients acceptable symptom state; PASS: patient acceptable symptom state; SJC28: swollen joint count (28); TJC28: tender joint count (28); VAS: visual analogue scale.

Of these patients, 572 (50.8%) were in PASS, whereas 555 (49.2%) were N-PASS. Patients in PASS were older than N-PASS patients at baseline [median (IQR) age: PASS = 63 (51, 70) years; N-PASS = 59 (49, 68) years, *P* = 0.0002] and the PASS group had a lower proportion of women than the N-PASS group [*N* (%) women: PASS = 343 (60.0); N-PASS = 371 (66.9), *P* = 0.017]. Furthermore, patients in the PASS group had statistically significantly lower scores on all clinical and patient-reported outcomes measured at baseline, apart from the proportion of patients who were RF+, which did not differ between the PASS groups (Table 1). Multivariable analysis indicated that the baseline variables independently associated with increased odds of being in PASS at baseline were: older age [OR per year increase 1.01 (95% CI 1.00, 1.02)], shorter disease duration [OR per month increase 0.96 (95% CI 0.94, 0.99)], lower SJC28 [OR per swollen joint increase 0.97 (95% CI 0.94, 1.00)], lower HAQ score [OR per unit increase in HAQ 0.73 (95% CI 0.54, 0.98)], lower pain-VAS [OR per standard deviation increase 0.43 (95% CI 0.35, 0.53)], lower fatigue-VAS [OR per standard deviation increase 0.74 (95% CI 0.60, 0.90)] and lower HADS-depression [OR per unit increase 0.92 (95% CI 0.86, 0.97)] (supplementary Table S3, available at *Rheumatology* online). The PASS and N-PASS groups did not differ in terms of oral or i.m. steroid use.

### Phenotypes within patients in PASS at baseline

K-medians cluster analysis was used to identify clinical phenotypes within the group in PASS at baseline. Among the 572 patients in PASS at baseline, six distinct clusters were identified and ordered in terms of severity score, so that Cluster 1 represented the least severe cluster and Cluster 6 the most severe (Table 2).

**Table 2:** Baseline characteristics of the six clusters

		Baseline scores, median (IQR)						Clinical characteristics matrix^a^					
Cluster	*N*	SJC28	TJC28	HAQ	Pain	Fatigue	Depr	SJC28	TJC28	HAQ	Pain	Fatigue	Depr
1	113	2 (0, 4)	1 (0, 2)	0.00 (0.00, 0.25)	10 (3, 16)	6 (0, 13)	1 (0, 2)	M	L	L	L	L	L
2	159	2 (1, 5)	3 (1, 6)	0.50 (0.25, 0.75)	30 (21, 46)	38 (24, 54)	2 (1, 3)	M	M	M	M	M	M
3	118	3 (1.5, 6)	3 (1, 6)	0.88 (0.50, 1.38)	26 (18, 34)	31 (19, 48)	7 (5, 8)	M	M	M	M	M	H
4	65	12 (9, 17)	12 (6, 15)	0.50 (0.13, 0.88)	23 (10, 40)	19 (5, 32)	2 (1, 4)	H	H	M	M	M	M
5	71	3 (1, 6)	6 (2.5, 8)	1.50 (1.13, 1.88)	70 (55, 79)	68 (55, 80)	8 (4, 10)	M	M	H	H	H	H
6	46	11 (6, 13)	20 (14, 24)	1.56 (1.00, 2.00)	60 (45, 73)	69 (48, 78)	7 (4, 10)	H	H	H	H	H	H

a Low (L), medium (M) and high (H) defined based on 33rd and 66th centile of each characteristic in the total cohort of patients in PASS: SJC28: L = 0–1, M = 2–6, H = 7–28; TJC28: L = 0–1, M = 2–6, H = 7–28; HAQ: L = ≥0 and <0.26, M = ≥0.26 and ≤0.88, H = >0.88 and ≤3; pain: L = 0–18, M = 19–38, H = 39–100; fatigue: L = 0–17, M = 18–47, H = 48–100; depression: L = 0–1, M = 2–5, H = 6–14. Depr: depression, measured using the Hospital Anxiety and Depression Scale; IQR: interquartile range; *N*: number; SJC28: swollen joint count (28); TJC28: tender joint count (28).

Cluster 1 had low median scores of disease activity (SJC28, TJC28) and patient-reported outcome measures (PROMs: HAQ, pain-VAS, fatigue-VAS, HADS-depression) [*N* (% of total PASS group) =113 (19.7)] and Cluster 2 had moderate median scores across disease activity and PROMs [*N* (%) = 159 (27.8)]. Cluster 3 [*N* (%) = 118 (20.6)] had similar scores to Cluster 2 across disease activity and PROMs, except that Cluster 3 had high levels of depression [median (IQR) HADS-depression: Cluster 2 = 2 (1, 3); Cluster 3 = 7 (5, 8)]. Cluster 4 had high disease activity, but moderate PROMs [*N* (%) = 65 (11.4)], whereas Cluster 5 had moderate disease activity and high PROMs [*N* (%) = 71 (12.4)]. Cluster 6 had relatively high disease activity and PROMs across the measures [*N* (%) = 46 (8.0)]. Clusters 5 and 6 were slightly younger on average than the other clusters, and Clusters 5 and 6 had significantly more women. Higher proportions of Clusters 5 and 6 had received i.m. steroids in the week prior to baseline (supplementary Table S4, available at *Rheumatology* online).

### Outcomes over follow-up

In total, 862 patients were seen at the 6-month assessment and 700 patients were seen at 12 months. Patients in PASS at baseline had consistently better outcomes at 6 and 12 months compared with N-PASS patients (Table 3). Over one year, patients in PASS at baseline had on average lower HAQ scores [mean difference −0.48 (95% CI −0.56, −0.41)], lower DAS28 scores [mean difference −0.71 (95% CI −0.83, −0.59)], lower pain-VAS scores [mean difference −17.6 (95% CI −20.0, −15.3)], lower fatigue-VAS scores [mean difference −17.1 (95% CI −19.8, −14.3)], and lower HADS-depression and HADS-anxiety scores [mean difference: depression −2.3 (95% CI −2.7, −1.9); anxiety −1.8 (95% CI −2.2 to −1.3)] compared with baseline N-PASS patients. Furthermore, being in PASS at baseline was strongly associated with being in PASS over follow-up [PASS *vs* N-PASS at baseline: OR 19.7 (95% CI 15.7, 24.7)].

**Table 3:** Six- and 12-month outcome, stratified by baseline PASS status

	6 months					12 months				
	BL PASS		BL N-PASS			BL PASS		BL N-PASS		
Outcome	*N*	Median (IQR)	*N*	Median (IQR)	P	*N*	Median (IQR)	*N*	Median (IQR)	P
SJC28	429	1 (0, 2)	433	2 (0, 5)	<0.0001^a^	336	0 (0, 2)	364	1 (0, 4)	0.0054^a^
TJC28	428	1 (0, 4)	436	3 (1, 8)	<0.0001^a^	336	1 (0, 4)	363	2 (0, 8)	<0.0001^a^
DAS28	411	2.7 (1.9, 3.6)	417	3.4 (2.5, 4.5)	<0.0001^a^	323	2.7 (1.9, 3.5)	346	3.1 (2.2, 4.2)	<0.0001^a^
HAQ	401	0.50 (0.00, 1.00)	376	1.00 (0.38, 1.63)	<0.0001^a^	343	0.50 (0.00, 1.00)	317	0.88 (0.25, 1.50)	<0.0001^a^
Pain-VAS	397	20 (8, 43)	373	31 (16, 58)	<0.0001^a^	338	20 (8, 37)	315	28 (12, 51)	<0.0001^a^
Fatigue-VAS	394	28 (12, 59)	373	50 (23, 72)	<0.0001^a^	340	26 (9, 51)	313	45 (19, 68)	<0.0001^a^
HADS-depression	401	3 (1, 6)	375	5 (2, 8)	<0.0001^a^	343	2 (1, 6)	313	5 (2, 8)	<0.0001^a^
HADS-anxiety	400	4 (1, 7)	372	6 (3, 9)	<0.0001^a^	344	4 (1.5, 7)	313	5 (2, 9)	<0.0001^a^
PASS, *N* (%)	330 (84.4) [missing: 71]		224 (61.2) [missing: 88]		<0.001^b^	292 (86.4) [missing: 56]		211 (70.3) [missing: 90]		<0.001^b^

a Mann–Whitney U test. ^b^Chi^2^ test. BL: baseline; HADS: Hospital Anxiety and Depression Scale; IQR: interquartile range; *N*: number; N-PASS: not in a patient acceptable symptom state; PASS: patient acceptable symptom state; SJC28: swollen joint count (28); TJC28: tender joint count (28); VAS: visual analogue scale.

**Fig. 1:**
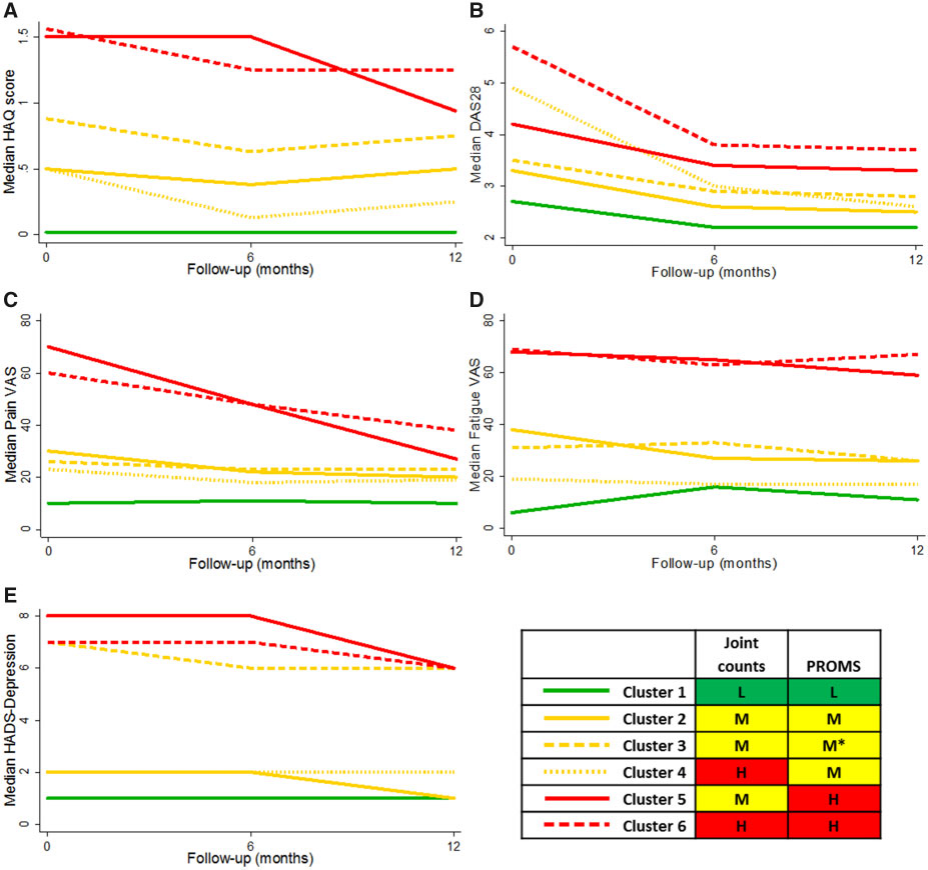
Outcomes over 12 months of follow-up, stratified by cluster (**A**) HAQ, (**B**) DAS28, (**C**) pain-VAS, (**D**) fatigue-VAS and (**E**) HADS-depression. *Moderate level PROMs other than HADS-depression, which was high. H: high; HADS: Hospital Anxiety and Depression Scale; L: low; M: moderate; PROMs: patient-reported outcome measures; VAS: visual analogue scale.

Table 4 contains the outcomes of the patients in PASS over 12 months, stratified by cluster. HAQ scores were low for Cluster 1 (low disease activity, low PROMs), Cluster 2 (moderate disease activity, moderate PROMs) and Cluster 4 (high disease activity, moderate PROMs). HAQ scores were moderate for patients in Cluster 3 (moderate disease activity, moderate PROMs apart from high depression). Clusters 5 (moderate disease activity, high PROMs) and 6 (high disease activity, high PROMs) had high HAQ scores over 12 months (Fig. 1A). DAS28 at 6 and 12 months increased sequentially as cluster severity increased. Cluster 5 had higher DAS28 scores at 6 and 12 months compared with Cluster 4, despite Cluster 4 being characterized by high disease activity at baseline and Cluster 5 having moderate disease activity (measured using SJC28 and TJC28) (Fig. 1B). Pain-VAS and fatigue-VAS were low at 6 and 12 months for Clusters 1–4, and high for Clusters 5 and 6 (Fig. 1C and D). A similar pattern was seen for HADS-depression scores, except that Cluster 3 continued to have high levels of depression over follow-up (Fig. 1E).

**Table 4:** Outcomes over follow-up, stratified by cluster

	Cluster, median (IQR)					
	1	2	3	4	5	6
Outcome						
HAQ, 6 months	0.06 (0.00, 0.38)	0.38 (0.06, 0.88)	0.63 (0.25, 1.13)	0.13 (0.00, 0.63)	1.5 (0.88, 1.88)	1.25 (0.88, 1.63)
HAQ, 12 months	0.00 (0.00, 0.38)	0.50 (0.00, 0.88)	0.75 (0.25, 1.19)	0.25 (0.00, 0.63)	0.94 (0.50, 1.63)	1.25 (0.75, 1.63)
DAS28, 6 months	2.2 (1.7, 2.7)	2.6 (1.8, 3.4)	2.9 (2.3, 3.6)	3.0 (2.0, 4.1)	3.4 (2.2, 4.2)	3.8 (2.8, 4.9)
DAS28, 12 months	2.2 (1.8, 3.2)	2.5 (1.9, 3.4)	2.8 (2.2, 3.3)	2.6 (1.9, 3.6)	3.3 (2.4, 3.6)	3.7 (3.1, 4.4)
Pain-VAS, 6 months	11 (4, 19)	22 (11, 37)	23 (11, 37)	18 (7, 32)	48 (25, 71)	48 (24, 68)
Pain-VAS, 12 months	10 (2, 27)	20 (11, 32)	23 (11, 41)	19 (8, 33)	27 (16, 65)	38 (24, 51)
Fatigue-VAS, 6 months	16 (4, 27)	27 (10, 51)	33 (21, 54)	17 (8, 46)	65 (44, 74)	63 (41, 71)
Fatigue-VAS, 12 months	11 (2, 34)	26 (9, 48)	26 (12, 47)	17 (9, 37)	59 (26, 77)	67 (45, 78)
HADS-depression, 6 months	1 (1, 4)	2 (1, 4)	6 (4, 8)	2 (1, 4)	8 (4, 10)	7 (3, 9)
HADS-depression, 12 months	1 (0, 4)	1 (1, 3)	6 (3, 9)	2 (1, 4)	6 (1, 8)	6 (2, 10)

	Cluster, *N* (% of cluster)					
	1	2	3	4	5	6
Outcome						
PASS, 6 months	77 (94)	95 (87)	69 (84)	44 (90)	26 (62)	19 (70)
PASS, 12 months	58 (84)	78 (84)	64 (91)	44 (90)	24 (83)	24 (86)

	Cluster, mean difference (95% CI)^a^					
	1	2	3	4	5	6
Outcome, over the repeated measures						
HAQ	0 (ref)	0.21 (0.08, 0.35)	0.44 (0.30, 0.59)	0.11 (−0.06, 0.28)	1.01 (0.83, 1.19)	0.98 (0.78, 1.18)
DAS28	0 (ref)	0.42 (0.13, 0.72)	0.61 (0.29, 0.93)	0.71 (0.35, 1.06)	1.02 (0.63, 1.41)	1.46 (1.04, 1.88)
Pain-VAS	0 (ref)	10.3 (4.9, 15.7)	12.0 (6.3, 17.8)	7.9 (1.3, 14.6)	27.3 (20.2, 34.4)	26.2 (18.3, 34.1)
Fatigue-VAS	0 (ref)	10.5 (4.4, 16.6)	16.7 (10.2, 23.2)	4.8 (−2.7, 12.4)	34.3 (26.3, 42.4)	35.4 (26.5, 44.4)
HADS-depression	0 (ref)	0.44 (−0.35, 1.23)	3.75 (2.91, 4.59)	0.37 (−0.60, 1.35)	4.35 (3.33, 5.38)	3.94 (2.77, 5.11)

	Cluster, OR (95% CI)^a^					
	1	2	3	4	5	6
Outcome, over the repeated measures						
PASS	0 (ref)	0.7 (0.3, 1.4)	0.8 (0.34, 1.6)	1.0 (0.4, 2.6)	0.3 (0.1, 0.6)	0.4 (0.2, 1.0)

a Regression analysis controlling for age and gender. HADS: Hospital Anxiety and Depression Scale; IQR: interquartile range; OR: odds ratio; PASS: patient acceptable symptom state: VAS: visual analogue scale.

Clusters 1, 2 and 4 had lower scores than baseline N-PASS patients in terms of HAQ, DAS28, pain-VAS, fatigue-VAS and HADS-depression (supplementary Table S5, available at *Rheumatology* online). Cluster 3 had lower scores than the N-PASS patients on all outcomes, other than depression scores, which were comparable over follow-up. Clusters 5 and 6 had similar scores to N-PASS patients over follow-up across outcomes; in most cases, Cluster 5 had higher scores than N-PASS patients over follow-up [mean outcome (95% CI) over follow-up Cluster 5 *vs* N-PASS: HAQ 0.30 (0.12, 0.48); DAS28 −0.10 (−0.47, 0.26); pain-VAS 6.9 (0.2, 13.6); fatigue-VAS 9.6 (2.0, 17.1); HADS-depression 0.9 (−0.1, 1.9); Cluster 6 *vs* N-PASS: HAQ 0.26 (0.04, 0.48); DAS28 0.33 (−0.06, 0.72); pain-VAS 5.7 (−2.0, 13.4); fatigue-VAS 10.1 (1.4, 18.8); HADS-depression 0.5 (−0.7, 1.7)].

## Discussion

This is the first paper to characterize the one-year outcomes of patients with early RA commencing MTX who reported being in an acceptable symptom state at baseline. Just over half of early RA patients starting MTX treatment reported being in an acceptable state. These patients were older, more often male and had lower disease activity and PROMs compared with patients reporting not being in an acceptable state. These patients also had lower disease activity and PROMs over follow-up, compared with N-PASS patients. However, despite these PASS patients having improved outcomes on average compared with N-PASS patients, we have identified phenotypes within the PASS group that had poor outcomes. The clusters with the worst outcomes (Clusters 5 and 6) both had high PROMs, indicating the importance of these factors in predicting outcome. Indeed, the cluster with high disease activity and moderate PROMs (Cluster 4) did much better than Clusters 5 and 6 on all outcomes, including DAS28. The patients in Clusters 5 and 6 did worse than the baseline N-PASS patients over follow-up. Clusters 5 and 6, despite reporting being in an acceptable state, may require additional monitoring and support, and this analysis demonstrates the importance of PROMs in identifying these patients.

The proportion of patients in PASS at baseline in this cohort was high (50.8%), considering patients had not yet started treatment. The PASS was originally designed to provide cut-points for different outcomes based on the level that patients thought was ‘acceptable’, which could be used in clinical trials [19]. The high proportion of patients in PASS at baseline questions the utility of PASS to define cut-points for good response in early RA. This high proportion of early RA patients in PASS may reflect the drive to see patients early and get them onto therapy as soon as possible, within the ‘window of opportunity’ [20–22]. Patients may be going onto therapy before their condition has deteriorated to a level that is no longer acceptable.

After 12 months, a large proportion of patients (78.8%) were in PASS, indicating successful treatment. Studies of prevalent cases reported slightly lower proportions of patients in PASS compared with this study, although there was significant heterogeneity. For instance, the NOR-DMARD study (mean disease duration 7.6 years) reported that 40.9% of patients were in PASS at baseline [5]; a multicentre study across 10 European countries (mean disease duration 12.6 years) reported that 60.5% of patients considered themselves in PASS [7]; and an international study across seven countries (mean disease duration 10 years) reported that 70% of their cohort was in PASS after 4 weeks of follow-up [19].

Patients in PASS had consistently lower scores in all disease activity measures and PROMs at baseline compared with N-PASS patients, consistent with other studies [7], and these lower scores continued over one year. Therefore, it is clear that PASS is, on average, a marker for good outcome. However, we have demonstrated that there are six symptom clusters in patients who report being in PASS at baseline, ranging from very low disease activity and PROMs up to high disease activity and PROMs. Clusters 5 and 6 had high PROMs at baseline and went on to have poor outcomes over the course of 12 months, as bad as or worse than N-PASS patients. Each patient will have a threshold for what they consider acceptable and another for when they will seek healthcare. Sheppard *et al.* reported that symptom evaluation was a key factor in prompting patients to seek healthcare [23]. Perhaps when considering each symptom separately, patients in Clusters 5 and 6 rated these symptoms highly, even if they considered their overall health to be acceptable. The individual symptom (e.g. high hand joint pain) prompted them to seek healthcare, rather than their acceptable overall health assessment. Other factors potentially explaining the high PROMs in Clusters 5 and 6 include being unaware that their condition could be improved, understanding that their particular problems may not be amenable to medical treatment, fear of medication or perhaps misunderstanding the question.

When assessing the outcomes of these subgroups, the divergent DAS28 scores of Clusters 4 and 5 over follow-up is of particular interest. Cluster 4 had higher DAS28 at baseline but then the DAS28 score for this cluster dropped to levels similar to Clusters 1, 2 and 3, whereas the DAS28 scores of Cluster 5 did not fall as steeply and were similar to Cluster 6 by 12 months. This is likely because these two clusters scored highly on different aspects of the DAS28, both resulting in high composite scores at baseline (with Cluster 4 scoring highly on the SJC28, TJC28 and CRP, whereas Cluster 5 scored highly on the patient global-VAS). When the DAS28 was split into the two-component DAS28 [24], tender joint count and patient global-VAS, this appears to be the case (supplementary Tables S6 and S7, and supplementary Fig. S2, available at *Rheumatology* online). The aspects that Cluster 4 scored highly on may be more amenable to treatment than those for Cluster 5, hence the divergence [25, 26]. Scoring highly on the PROM aspect of the DAS28 is associated with future pain [27], which may also be demonstrated here as the non-DAS28 outcomes of Cluster 5 are also worse compared with Cluster 4.

This analysis has a number of strengths. This is the first longitudinal study to report on the outcome over 12 months of patients reporting being in PASS at baseline, building on the previous cross-sectional research. The large sample size allows identification of multiple clusters within the group of patients reporting being in PASS at baseline, with each cluster having sufficient numbers of patients to study outcomes. Limitations of this study include some loss to follow-up, typical of all observational research. Patients leaving the cohort were slightly younger and slightly more likely to be in PASS (data not shown). However, as patients did not differ on other important clinical factors (e.g. DAS28, HAQ), this analysis is unlikely to be heavily biased by loss to follow-up. Furthermore, the use of longitudinal models mean that the results are not biased if the data are missing at random [28]. In addition, there were some missing data on baseline covariates; multiple imputation was used to account for these missing data. A further limitation is the potential that different patients interpreted the PASS question differently. However, the question emphasized that patients were to report on their current condition, rather than any future potential condition, so variance between patients’ interpretations of the question should be minimal.

In conclusion, we have demonstrated that 50.8% of patients with early RA considered themselves in an acceptable state prior to commencing their first conventional synthetic DMARD, calling into question the utility of PASS-based outcomes in randomized trials of early RA patients. These patients have better outcomes than N-PASS patients over 12 months, indicating that PASS is a useful marker for overall outcome. Lastly, within this group of patients who reported being in an acceptable state at baseline, there are clusters of symptoms that were associated with poor outcomes over follow-up. These patients may require additional observation, particularly if they themselves are happy with their condition but are unable to forecast their future outcome.

## Supplementary Material

kez497_Supplementary_Data
