# Supplementary material for: Long-term outcomes of patients who rate symptoms of rheumatoid arthritis as ‘satisfactory’
Source: Rheumatology (Oxford). 2019 Nov 15;59(8):1853–61. doi: 10.1093/rheumatology/kez497 (PMC7382599; doi:10.1093/rheumatology/kez497)
Supplement: kez497_Supplementary_Data [file kez497_supplementary_data.zip › kez497-suppl_data/rhe-19-0859-File004.docx]

**SUPPLEMENTARY MATERIAL**

**Supplementary Table S1 – Severity score of each cluster**

| Cluster | Severity Score |
| --- | --- |
| 1 | 116 |
| 2 | 240 |
| 3 | 291 |
| 4 | 290 |
| 5 | 393 |
| 6 | 457 |

**Calculating Severity Score**

The severity score was calculated by ranking patients' scores for each variable used to perform clustering from least to most severe. The mean of the ranks of the six variables was calculated for each patient. Then the mean rank was calculated for each cluster. This was done for each of the 10 imputed datasets. Therefore, the average rank across the 6 variables is 116 for patients in cluster 1 (out of 572) whereas the average rank across the 6 variables for cluster 6 is 457. Whilst there is clear differentiation between most of the clusters - clusters 3 and 4 scored almost identically. They were numbered as such, because cluster 3 is closer in characteristics to cluster 2, whereas cluster 4 is opposite in characteristics to cluster 5. As these are the key comparisons in the paper, the clusters were numbered in for easier reading.

**Supplementary Table S2 – Number of imputations within each person that disagreed with the modal cluster [6 clusters]**

| Number disagreeing with modal cluster | N | % |
| --- | --- | --- |
| 0 | 279 | 48.8 |
| 1 | 177 | 30.9 |
| 2 | 40 | 7.0 |
| 3 | 27 | 4.7 |
| 4 | 26 | 5.6 |
| 5 | 19 | 3.3 |
| 6 | 4 | 0.7 |

Supplementary Table 2 shows the number of imputations per person in which the cluster algorithm assigned a different cluster than that person’s modal cluster value. The majority of patients had perfect or near perfect agreement in their cluster assignment. There were a few patients were there was substantial disagreement between the different imputed datasets. These are likely to be patients with large amounts of missing data across the variables used in the cluster analysis. However, the numbers are relatively small and therefore will not alter the analysis substantially.

**Supplementary Table S3 – multivariable baseline predictors of baseline PASS**

| Predictor | OR (95% CI) |
| --- | --- |
| Age, years | 1.01 (1.00, 1.02) |
| Women vs men | 1.03 (0.74, 1.43) |
| Disease duration, months | 0.96 (0.94, 0.99) |
| Smoking  Current smoker vs. Never smoker  Ex-smoker vs. Never smoker | 1.10 (0.72, 1.67)  0.83 (0.59, 1.17) |
| SJC28 | 0.97 (0.94, 1.00) |
| TJC28 | 1.02 (0.99, 1.04) |
| CRP | 1.00 (0.99, 1.00) |
| HAQ | 0.73 (0.54, 0.98) |
| RF+ vs RF- | 1.11 (0.78, 1.57) |
| VAS pain  Natural scale  Standardised scale | 0.97 (0.96, 0.98)  0.43 (0.35, 0.53) |
| VAS fatigue  Natural scale  Standardised scale | 0.99 (0.98, 1.00)  0.74 (0.60, 0.90) |
| VAS patient global  Natural scale  Standardised scale | 1.00 (0.99, 1.01)  0.90 (0.71, 1.15) |
| VAS physician global  Natural scale  Standardised scale | 0.99 (0.98, 1.00)  0.84 (0.66, 1.06) |
| HADS depression | 0.92 (0.86, 0.97) |
| HADS anxiety | 1.03 (0.98, 1.08) |

Anti-CCP: anti-cyclic citrullinated peptide antibody; CI: confidence interval; CRP: C-reactive protein; HADS: Hospital Anxiety and Depression Scales; HAQ: Health Assessment Questionnaire; OR: odds ratio; PASS: patient acceptable symptom state; RF: rheumatoid factor; SJC28: swollen joint count 28; TJC28: tender joint count 28; VAS: visual analogue scale

**Supplementary Table S4 – Extra characteristics of the clusters at baseline, median(IQR)**

|  | Cluster 1 | Cluster 2 | Cluster 3 | Cluster 4 | Cluster 5 | Cluster 6 | p |
| --- | --- | --- | --- | --- | --- | --- | --- |
| Age, years | 62 (51, 70) | 62 (51, 71) | 64.5 (56, 72) | 65 (53, 73) | 58 (47, 69) | 58.5 (46, 69) | 0.0248 § |
| Women, N(%) | 55 (48.7) | 98 (61.6) | 68 (57.6) | 36 (55.4) | 49 (69.0) | 37 (80.4) | 0.004 † |
| Symptom duration, months | 5 (3, 8) | 6 (3, 11) | 6.5 (4, 10) | 6 (3, 9) | 6 (3, 9) | 5 (3, 8) | 0.1378 § |
| Oral steroids, N(%) | 26 (23.0) | 45 (28.7) | 32 (27.4) | 14 (21.9) | 13 (18.3) | 8 (17.4) | 0.404 † |
| Intramuscular steroids, N(%) | 17 (15.5) | 35 (22.7) | 23 (20.0) | 13 (20.6) | 24 (34.8) | 14 (31.8) | 0.038 † |

§ = Kruskall-Wallis test, † = Chi^2^ test

**Supplementary Table S5 – Comparison of clusters against N-PASS patients at baseline**

|  | Mean difference [95% CI] | | | | | |  |
| --- | --- | --- | --- | --- | --- | --- | --- |
| Outcome, over the repeated measures | **N-PASS** | **Cluster 1** | **Cluster 2** | **Cluster 3** | **Cluster 4** | **Cluster 5** | **Cluster 6** |
| HAQ | 0 [ref] | -0.70  [-0.84, -0.55] | -0.50  [-0.62, -0.38] | -0.25  [-0.39, -0.12] | -0.59  [-0.76, -0.42] | 0.30  [0.12, 0.48] | 0.26  [0.04, 0.48] |
| DAS28 | 0 [ref] | -1.11  [-1.38, -0.84] | -0.70  [-0.93, -0.46] | -0.51  [-0.77, -0.24] | -0.41  [-0.73, -0.09] | -0.10  [-0.47, 0.26] | 0.33  [-0.06, 0.72] |
| Pain-VAS | 0 [ref] | -20.4  [-25.4, -15.5] | -10.1  [-14.5, -5.7] | -8.2  [-13.1, -3.2] | -12.4  [-18.6, -6.3] | 6.9  [0.2, 13.6] | 5.7  [-2.0, 13.4] |
| Fatigue-VAS | 0 [ref] | -24.7  [-30.4, -19.1] | -14.4  [-19.4, -9.4] | -8.3  [-13.9, -2.7] | -20.3  [-27.2, -13.3] | 9.6  [2.0, 17.1] | 10.1  [1.4, 18.8] |
| HADS-Depression | 0 [ref] | -3.5  [-4.2, -2.7] | -3.0  [-3.7, -2.3] | 0.4  [-0.4, 1.2] | -3.0  [-4.0, -2.1] | 0.9  [-0.1, 1.9] | 0.5  [-0.7, 1.7] |

**Supplementary Table S6 – Comparison of 2 component DAS28 between the clusters at each assessment**

|  | Cluster |  |  |  |  |  |
| --- | --- | --- | --- | --- | --- | --- |
|  | 1 | 2 | 3 | 4 | 5 | 6 |
| 2C-DAS28 at baseline | 2.3 (1.4, 3.1) | 2.4 (1.8, 3.2) | 2.8 (1.8, 3.6) | 4.9 (4.0, 5.7) | 3.1 (2.5, 4.0) | 4.3 (3.4, 5.3) |
| 2C-DAS28 at 6 months | 1.4 (0.8, 2.1) | 1.5 (0.8, 2.7) | 1.8 (1.2, 2.7) | 2.1 (0.8, 3.7) | 1.5 (1.0, 3.0) | 1.9 (0.7, 3.9) |
| 2C-DAS28 at 12 months | 1.1 (0.7, 2.1) | 1.6 (0.8, 2.4) | 1.7 (0.8, 2.6) | 2.1 (0.7, 3.1) | 2.4 (1.4, 3.4) | 1.7 (1.2, 3.1) |
| Random effects regression model | 0 [ref] | 0.27  (-0.05, 0.60) | 0.46  (0.11, 0.80) | 0.67  (0.29, 1.06) | 0.65  (0.23, 1.08) | 0.64  (0.19, 1.10) |

**Supplementary Table S7 – Comparison of TJC28 and patient global VAS between the clusters at each assessment**

|  | Cluster |  |  |  |  |  |
| --- | --- | --- | --- | --- | --- | --- |
|  | 1 | 2 | 3 | 4 | 5 | 6 |
| TJC28 at baseline | 1  (0, 2) | 3  (1, 6) | 3  (1, 6) | 12  (6, 15) | 6  (2.5, 8) | 20  (14, 24) |
| TJC28 at 6 months | 0  (0, 1) | 1  (0, 3) | 2  (0, 4) | 2  (0, 6) | 3  (1, 8.5) | 5.5  (2, 12.5) |
| TJC28 at 12 months | 1  (0, 2) | 1  (0, 3) | 2  (0, 4) | 1  (0, 4) | 2  (0.5, 5.5) | 4  (1, 10) |
| Global VAS at baseline | 13  [6, 20] | 22  [14, 38] | 30  [19, 44] | 27  [20, 50] | 47  [31, 62] | 50  [30, 65] |
| Global VAS at 6 months | 13  [5, 21] | 24  [11, 41] | 27  [15, 45] | 22  [10, 33] | 30  [19, 51] | 41  [30, 65] |
| Global VAS at 12 months | 12  [5, 30] | 20  [10, 31] | 22  [10, 42] | 17  [9, 28] | 24  [15, 36] | 50  [27, 67 |
| Random effects regression model [TJC28] | 0  [ref] | 1.1  [-0.1, 2.3] | 1.3  [0.0, 2.6] | 2.6  [1.2, 4.0] | 3.5  [1.9, 5.0] | 6.0  [4.4, 7.7] |
| Random effects regression model [Global VAS] | 0  [ref] | 9.7  [4.6, 14.5] | 11.6  [6.3, 17.0] | 5.1  [-1.0, 11.1] | 15.4  [9.1, 21.8] | 26.2  [19.2, 33.3] |

Median and IQR reported. Results from random effects models, controlling for age and gender are mean difference and 95% confidence interval. TJC28: tender joint count (28); VAS: visual analogue scale

**Supplementary figure legends**

**Supplementary Figure S1 - Flow chart of the selection of patients to be included in the study**

**Supplementary Figure S2 – Additional outcomes over time, stratified by PASS cluster 3** (a) 2C-DAS28, (b) tender joint count (28) and (c) patient global VAS stratified by cluster. 2C-DAS28: Disease Activity Score 28 (2 components); TJC28: tender joint count (28); VAS: visual analogue scale
