# Supplementary figures and images for: Long-term outcomes of patients who rate symptoms of rheumatoid arthritis as ‘satisfactory’
Source: Rheumatology (Oxford). 2019 Nov 15;59(8):1853–61. doi: 10.1093/rheumatology/kez497 (PMC7382599; doi:10.1093/rheumatology/kez497)

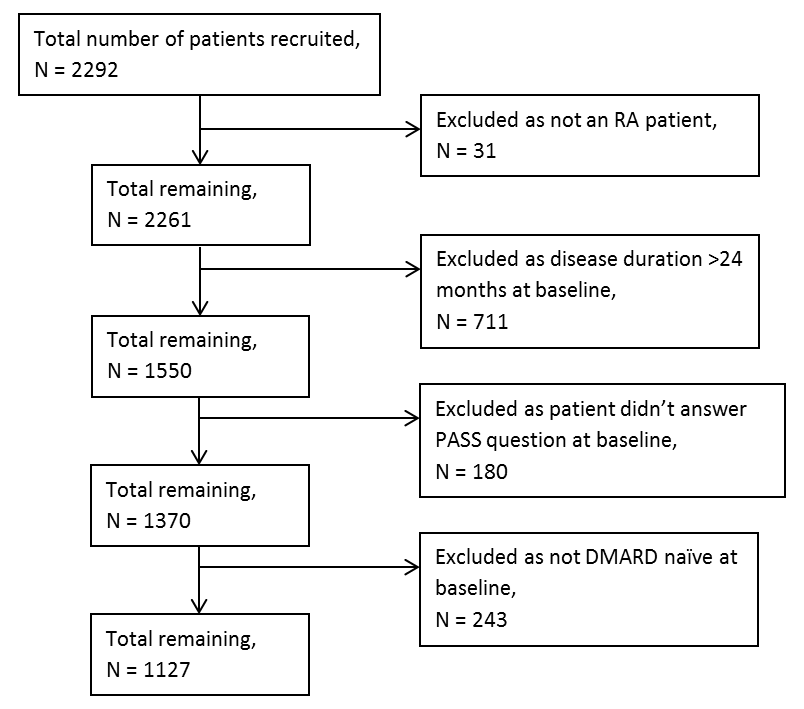

Supplement: kez497_Supplementary_Data [file kez497_supplementary_data.zip › kez497-suppl_data/rhe-19-0859-File005.tif]

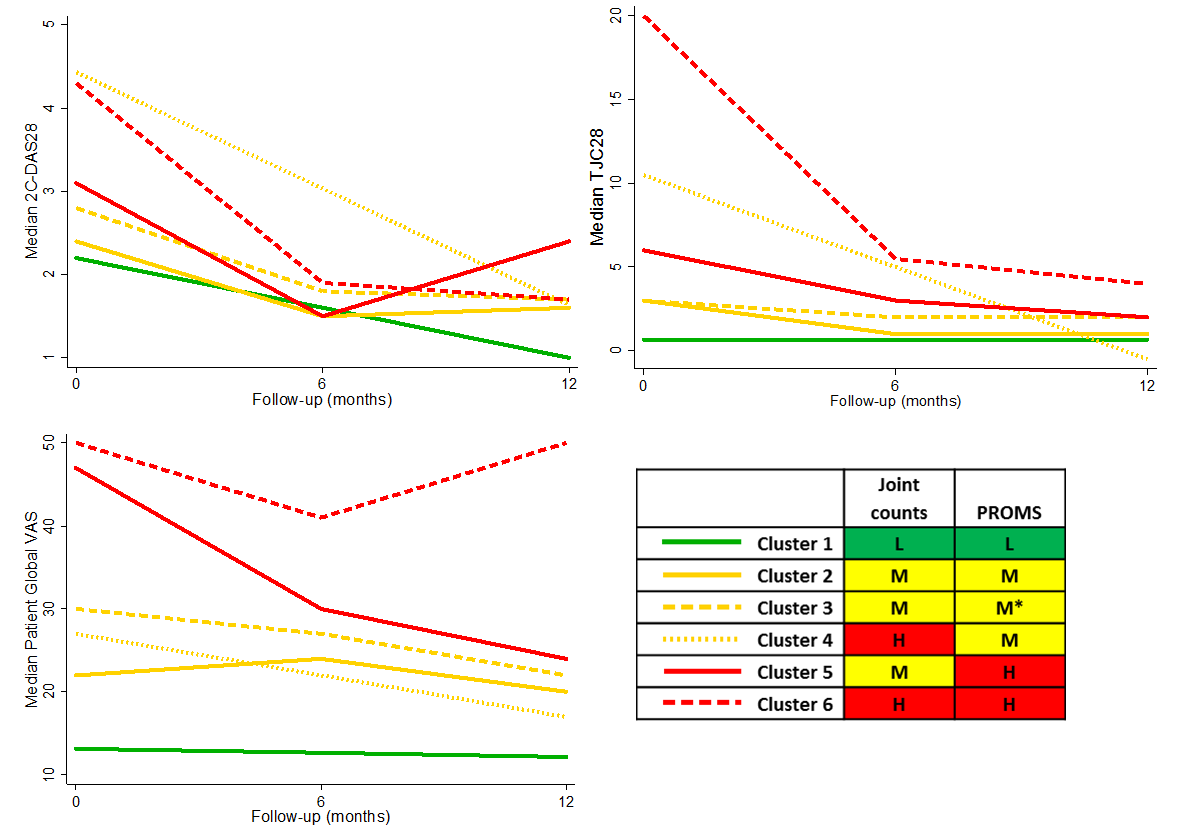

Supplement: kez497_Supplementary_Data [file kez497_supplementary_data.zip › kez497-suppl_data/rhe-19-0859-File006.tif]
